# Supplementary material for: A shape-driven reentrant jamming transition in confluent monolayers of synthetic cell-mimics
Source: Nat Commun. 2024 Jul 5;15:5645. doi: 10.1038/s41467-024-49044-z (PMC11226658; doi:10.1038/s41467-024-49044-z)
Supplement: Supplementary file 1 — Supplementary Information [file 41467_2024_49044_MOESM1_ESM.pdf]

Supplementary Material for
"A shape-driven reentrant jamming transition in confluent monolayers of synthetic cell-mimics"

Pragya Arora,[1] Souvik Sadhukhan,[2] Saroj Kumar Nandi,[2] Dapeng Bi,[3] A K Sood,[4,5] and Rajesh Ganapathy[5,6]

[1] *Chemistry and Physics of Materials Unit, Jawaharlal Nehru Centre for Advanced Scientific Research, Jakkur, Bangalore - 560064, INDIA*
[2] *Tata Institute of Fundamental Research, Hyderabad - 500046, INDIA*
[3] *Department of Physics, Northeastern University, Boston, MA 02115, U.S.A.*
[4] *Department of Physics, Indian Institute of Science, Bangalore- 560012, INDIA*
[5] *International Centre for Materials Science, Jawaharlal Nehru Centre for Advanced Scientific Research, Jakkur, Bangalore - 560064, INDIA*
[6] *School of Advanced Materials (SAMat), Jawaharlal Nehru Centre for Advanced Scientific Research, Jakkur, Bangalore - 560064, INDIA*

(Dated: May 15, 2024)

CONTENTS

I. Supplementary Methods 1
  A. Confinement of achiral polar active ellipsoids within paper rings 1
  B. Details of cell-mimic composition for the various activities 1

II. Supplementary Discussion 5
  A. Relaxation dynamics of confluent cell monolayers 5
  B. Quantifying clustering of active cells through the probability distribution of Voronoi cell areas 5
  C. Shape and shape variability in confluent cell-mimic monolayers 6
  D. Boundary effects 8
  E. Brief description of the mean-field theory of cell shape variability and the link between structure and dynamics 8
  F. Quantifying dynamical heterogeneity 10
  G. Numerical Methods 10
    1. Cellular potts model 11
    2. Vertex Model 11
  H. Fast cells violate mean-field predictions 12

III. Supplementary References 13

I. SUPPLEMENTARY METHODS

A. Confinement of achiral polar active ellipsoids within paper rings

In our initial experiments, we confined prolate active ellipsoids with a long axis measuring $\alpha = 6$ mm and shorter axes measuring $\beta = 2.4$ mm and $\delta = 2.1$ mm **Supplementary Fig. 4A**. During vertical vibration, the achiral polar active ellipsoids within the paper rings often aggregated at diametrically opposed ends due to the interplay between membrane curvature and particle orientation, with their polarity prominently oriented outward. This aggregation not only limited cell motility but also led to non-uniform cell stiffness **Supplementary Fig. S4B and C**.

B. Details of cell-mimic composition for the various activities

To tune the motility of the cell-mimics, we changed the handedness of a few ellipsoids inside the membrane from (−) to (+). The experimental procedure involved placing ellipsoids of the same handedness initially, followed by individually flipping a few ellipsoids within the paper rings to achieve the desired chirality within the cell interior,

$\chi_{\rm cell}$. Details about the count of ellipsoids for each handedness can be found in **Supplementary Table 1**, where N_+ and N_- represent the number of (+) and (−) ellipsoids, respectively.

χ_{cell}	N_+	N_-
1	20	0
0.9	19	1
0.8	18	2
0.6	16	4
0.4	14	6
0.2	12	8
0	10	10
-0.2	8	12
-0.4	4	16
-0.8	2	18
-0.9	1	19
-1	0	20

Supplementary Table. I: Changing the chirality of the cell interior

The dynamics of individual cells were quantified by performing experiments with one or two cells on the plate. This helps avoid cell-cell interactions. The translational mean-squared displacement, $\langle \Delta r^2 \rangle$, and speed distribution, $P(v)$, of the centre-of-mass (COM) of each cell were used to quantify the dynamics. **Supplementary Fig. 5A** shows the translational mean-squared displacement (MSD),

$$\langle \Delta r^2 \rangle = \frac{1}{N} \sum_{i=1}^{N} \langle \Delta r_i(t)^2 \rangle \tag{1}$$

at various $\chi_{\rm cell}$ for isolated cells. Here, $\langle\rangle$ denotes an average over the number of ellipsoids N, and $\Delta r_i(t)$, is the translational displacement of the ith cell. We identified the crossover time from ballistic to diffusive dynamics (shown by the dashed lines) as the persistence time, τ_p.

Supplementary Fig. 5B shows the probability distribution of speed $P(v)$ corresponding for a lag time of 1.25 s. The individual velocities are defined as:

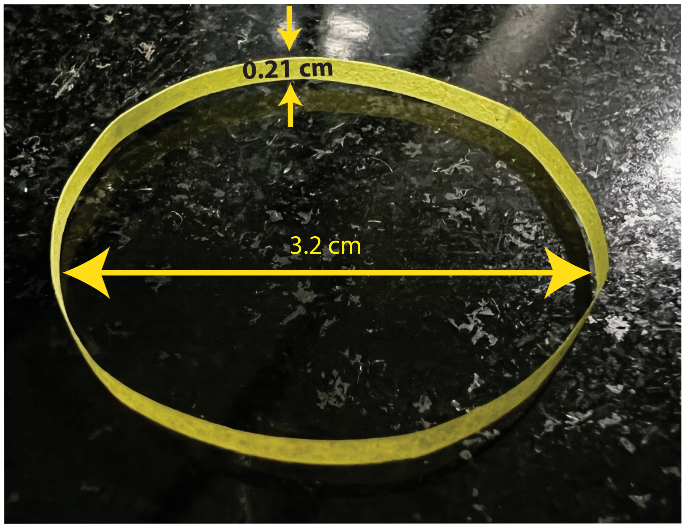

Supplementary Fig. 1: Snapshot of a flexible paper rings (3 cm in diameter) of thickness 40 μm.

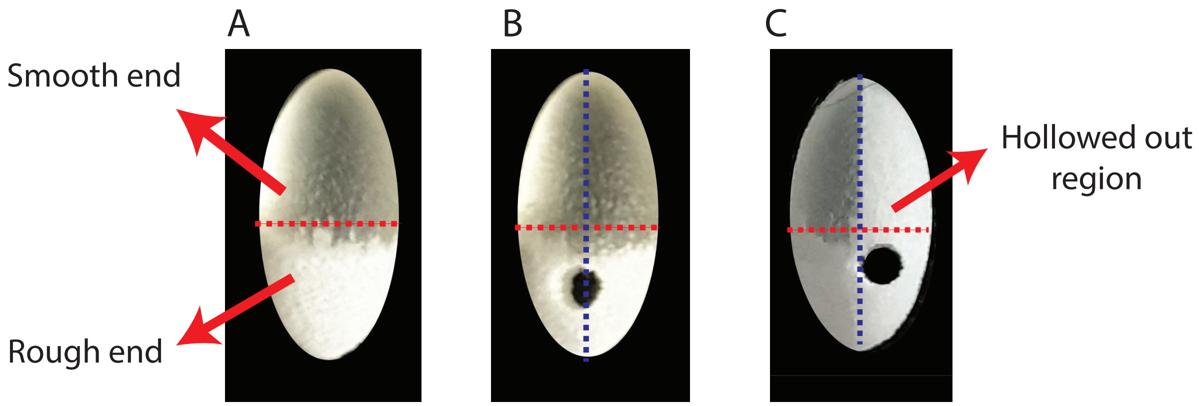

Supplementary Fig. 2: **(A)** Polar active ellipsoids with an asymmetry in friction coefficient μ.**(B)** Polar active ellipsoids with an asymmetry in μ and mass m. **(C)** Polar chiral active ellipsoids with left-right mass asymmetries. The red dashed lines show the hollowed-out portion of the ellipsoid.

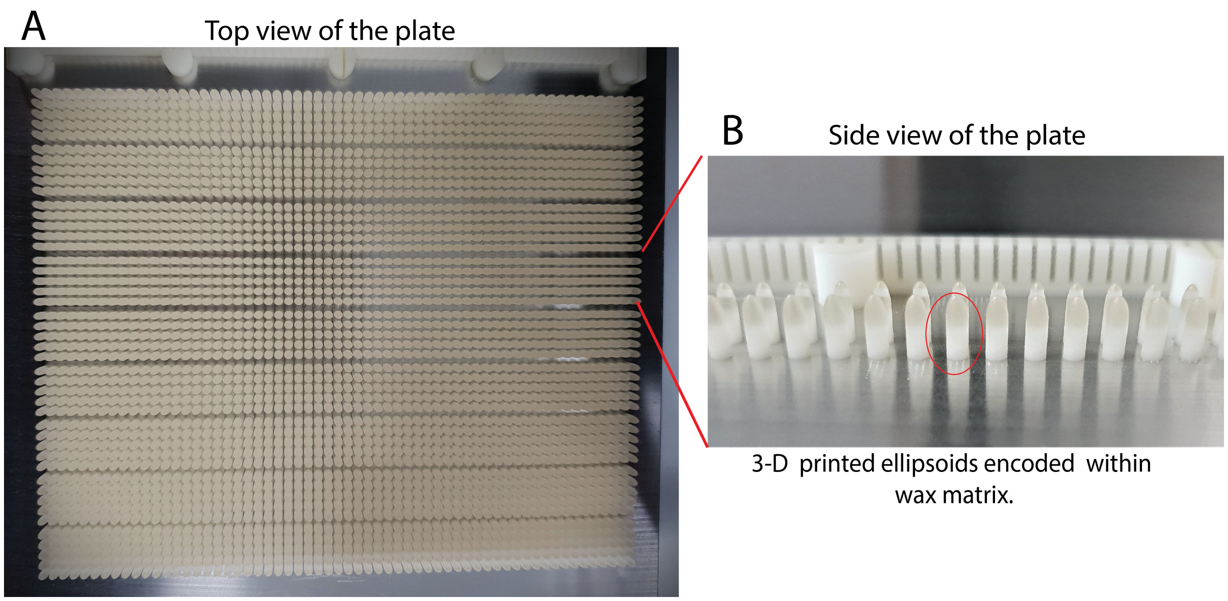

Supplementary Fig. 3: **(A)** Top view of tray with a batch of 3D printed particles. **(B)** Side view of tray with a batch of 3D printed particles.

$$\vec{v}_i(t) = \left[\vec{r}_i\left(t+\tau_0\right) - \vec{r}_i(t)\right]/\tau_0 \tag{2}$$

Notably, the persistence time of particles' directed motion, τ_p, and v exhibited a systematic increase with increasing $|\chi_{\rm cell}|$ values. As anticipated, tuning χ_{cell} even while keeping the total number of particles within the paper rings constant at $N = 20$ particles allows tuning the cell activity (**Supplementary Fig. 5A and B**).

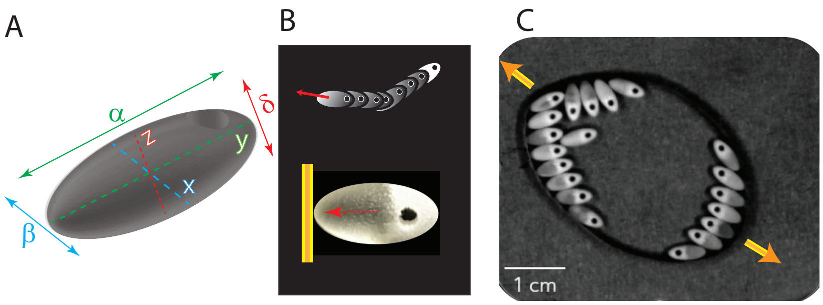

Supplementary Fig. 4: **(A)** 3D view of the ellipsoid showing its dimensions.**(B)** Due to a persistent force, achiral active particles align perpendicular to the walls. The red arrow indicates the direction of the self-propulsion. **(C)** The interplay of membrane curvature and particle orientation results in the accumulation of polar active ellipsoids at diametrically opposite ends with their polarity pointing outwards. Moreover, this results in low cell motility and nonuniform cell stiffness.

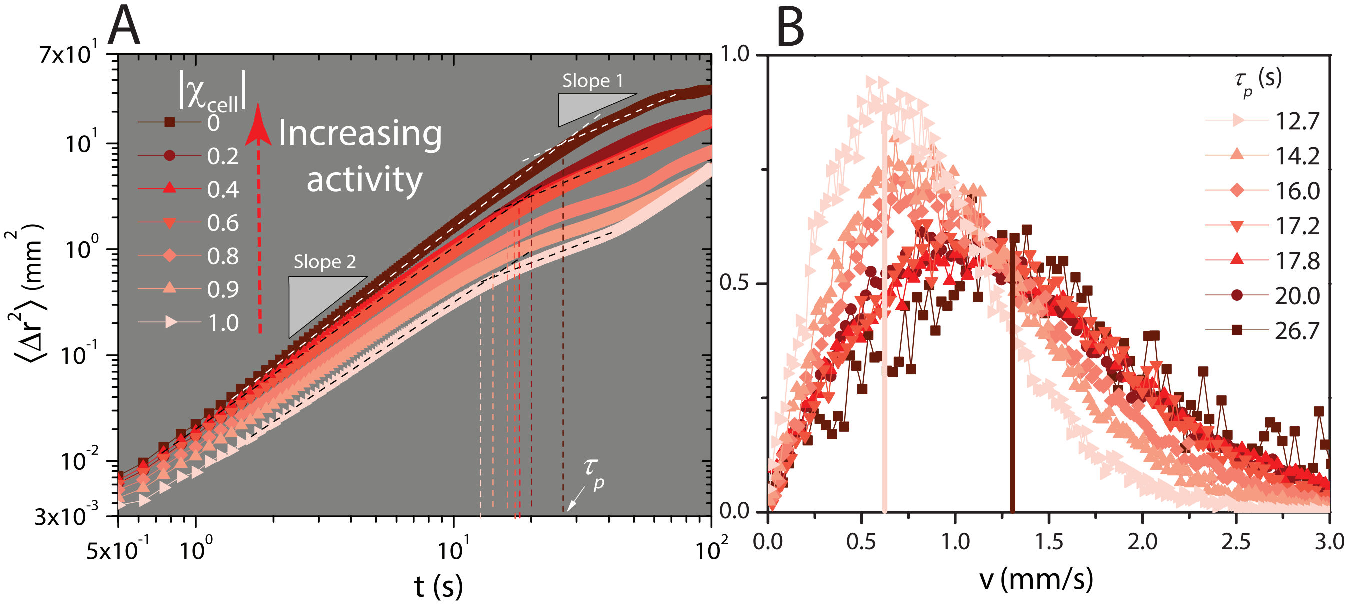

Supplementary Fig. 5: **(A)** Translational mean-squared displacement (MSD) $\langle \Delta r^2 \rangle$ versus lag time t for isolated cells ($\phi < 1\%$) for different $|\chi_{\rm cell}|$ values. **(B)** Probability distribution of individual membrane velocity v at $\phi = 0.1$. Solid vertical lines represent the mean speed of individual cells.

$\lvert\chi_{\rm cell}\rvert$	τ_p s	v mm/s	$l_p = v\tau_p$ mm
1	12.7	0.60	7.62
0.9	14.2	0.74	10.50
0.8	16.0	0.83	13.28
0.6	17.2	1.01	17.37
0.4	17.8	1.02	18.15
0.2	20.0	1.03	20.6
0	26.7	1.34	35.77

Supplementary Table. II: Details of various active granular cells

II. SUPPLEMENTARY DISCUSSION

A. Relaxation dynamics of confluent cell monolayers

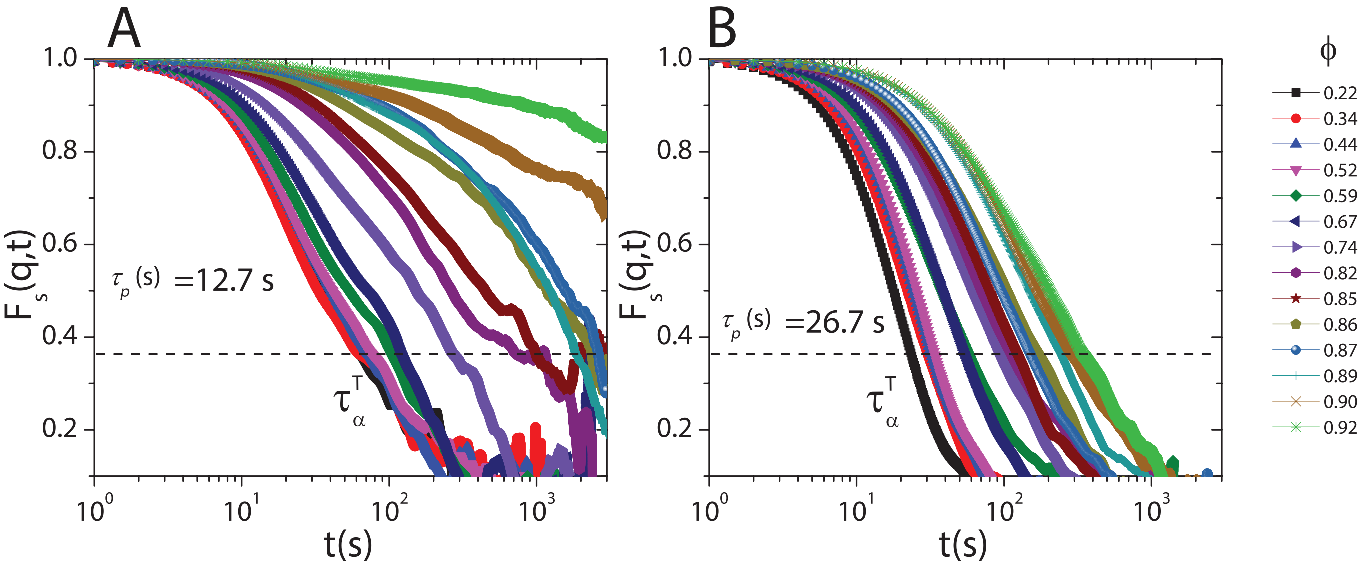

Supplementary Fig. 6: **(A, B)** Self intermediate scattering function $F_s(q = 2\text{cm}^{-1}, t)$ for various ϕ's for two representative activities.

The translational dynamics was quantified using the self-intermediate scattering function $F_s(q,t)$ defined as:

$$F_s(\mathbf{q},t) = \frac{1}{N}\left\langle \sum_{j=1}^{N} \exp\left(i\mathbf{q}\cdot\left(\mathbf{r}_j\left(t+t_0\right)-\mathbf{r}_j(t)\right)\right)\right\rangle \tag{3}$$

Here q is the wavevector, N is the total number of particles, and $r_j(t)$ is the position of the jth ellipsoid at time t. The translational relaxation times τ_α^T, were defined as the time at which the corresponding correlation functions decay to 1/e. The variation of $F_s(q,t)$ as a function of ϕ for for two representative activities is shown in (**Supplementary Fig. 6A and B**).

B. Quantifying clustering of active cells through the probability distribution of Voronoi cell areas

As the activity of the deformable synthetic granular membranes was increased at a constant ϕ, we observed a marked tendency for the particles to cluster together. This observation was quantified by analyzing the probability distribution of Voronoi cell areas, obtained from the membrane's centroid (**Supplementary Fig. 7A**). This enabled us to measure the local packing fraction. To achieve this, we tessellated the space around the membrane centers into Voronoi cells and subsequently estimated the probability distribution of Voronoi cell area (**Supplementary Fig. 7B**).

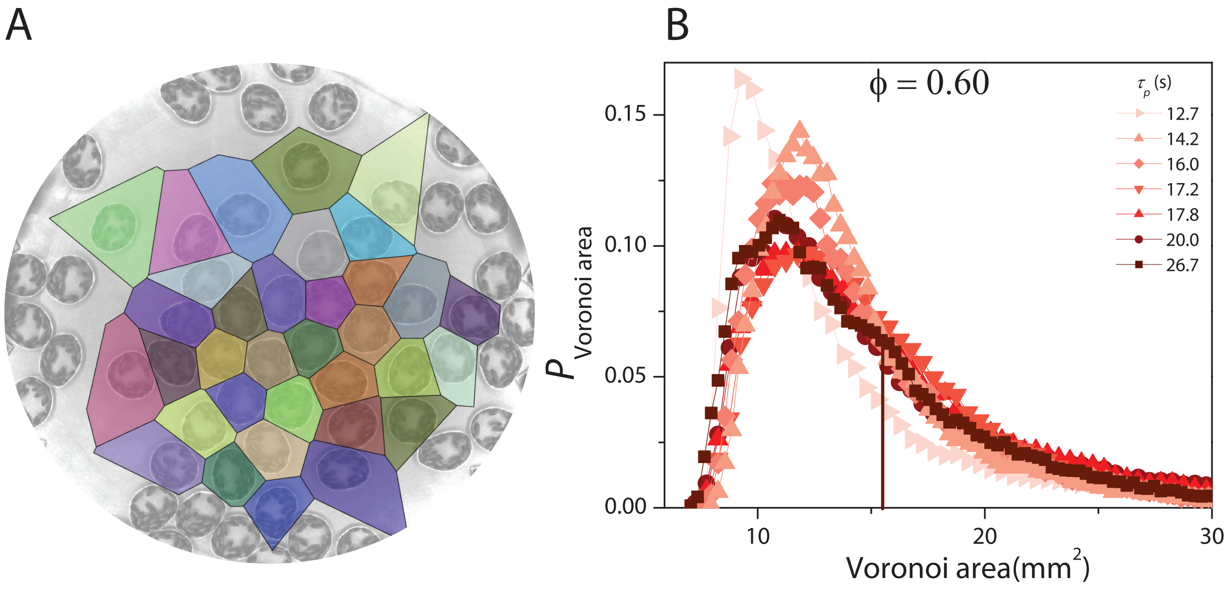

Supplementary Fig. 7: **(A)** Voronoi tessellation of the membrane centers to obtain local densities. **(B)** Probability distributions of Voronoi area exhibited a shoulder as activity increased, indicating a strong tendency for particles to cluster with increasing activity.

C. Shape and shape variability in confluent cell-mimic monolayers

The aspect ratio AR and the standard deviation of the aspect ratio $SD(AR)$ were used as metrics to quantify the shape and variability, respectively, of our granular cell assemblies. The relationship between $SD(AR)$ and $\overline{AR}$ for granular cells adheres to the same universal scaling observed in living systems [1], following the functional form ($SD(AR) \simeq 0.71\overline{AR} - 0.75$). The solid black line represents this universal scaling relation in (**Supplementary Fig. 8**)

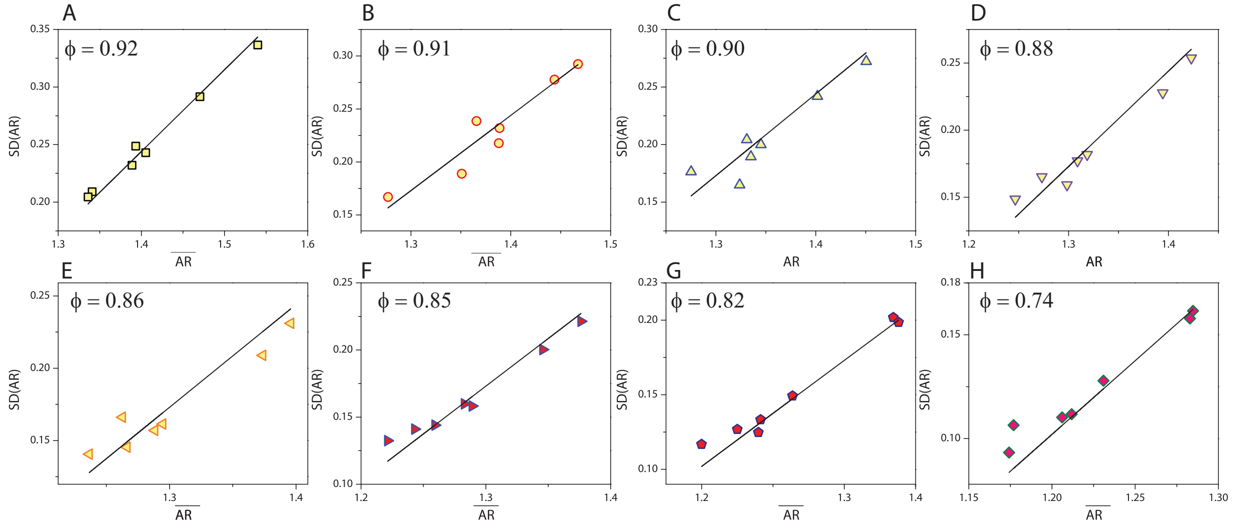

Supplementary Fig. 8: $SD(AR)$ vs the $\overline{\text{AR}}$ follows a universal relation ($SD(AR) \simeq 0.71\overline{AR} - 0.75$), the solid black line represents this universal scaling relation.

Subsequently, we analyzed the probability density functions (PDFs) of AR, noting that the PDF exhibited a broad distribution with a significant positive skew for the unjammed (fluid-like) state, which was observed at an intermediate value of $\tau_p = 20$ (refer to **Supplementary Fig. 9A**, **Supplementary Fig. 10A**, and **Supplementary Fig. 11A**).

Conversely, for the more jammed states corresponding to other τ_p values, the PDFs were narrower and displayed a smaller skew.

Furthermore, through the process of rescaling AR to $x = \frac{(AR-1)}{(\overline{AR}-1)}$, we observed the collapse of the PDFs at different τ_p values onto a single universal distribution. Within the experimental uncertainty, the scaled PDFs for all values of ϕ near confluence were equally well-fitted by the $k-$gamma distribution (4), with the parameter k ranging between 2.5 and 2.8 (refer to **Supplementary Fig. 9B**, **Supplementary Fig. 10B**, and **Supplementary Fig. 11B**).

$$P(x,k) = \left[k^k/\Gamma(k)\right] x^{k-1} \exp[-kx] \tag{4}$$

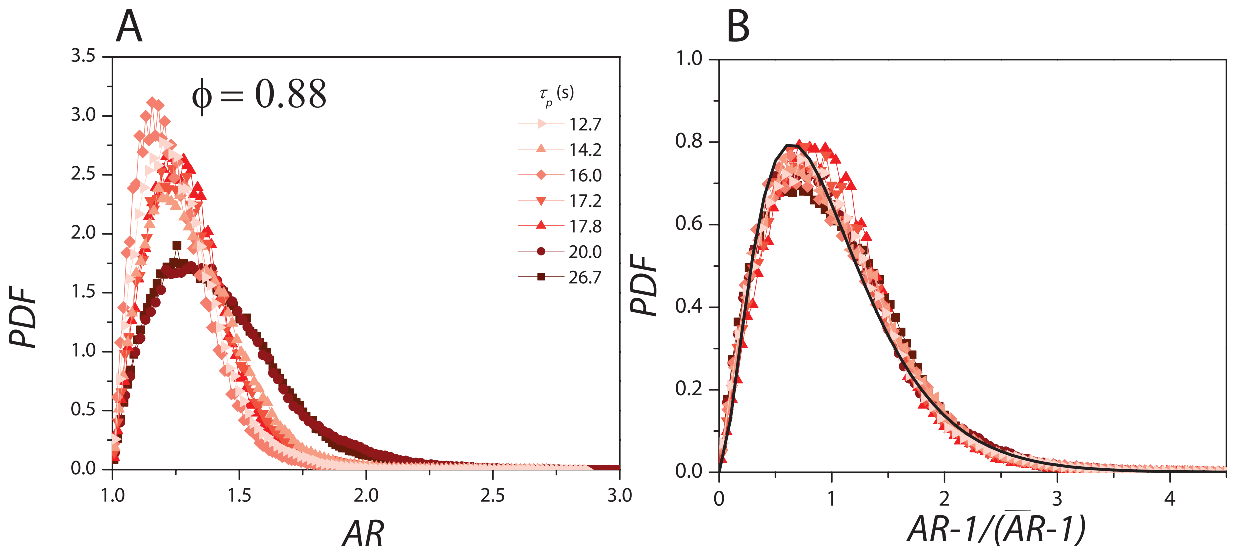

Supplementary Fig. 9: **(A)** Probability density functions (PDFs) of AR for different τ_p values for $\phi = 0.88$. **(B)** When scaled by $(AR-1)/(\overline{AR}-1)$, the PDF at different τ_p values collapse. The black line represents a $k-$Gamma distribution ($k = 2.8$).

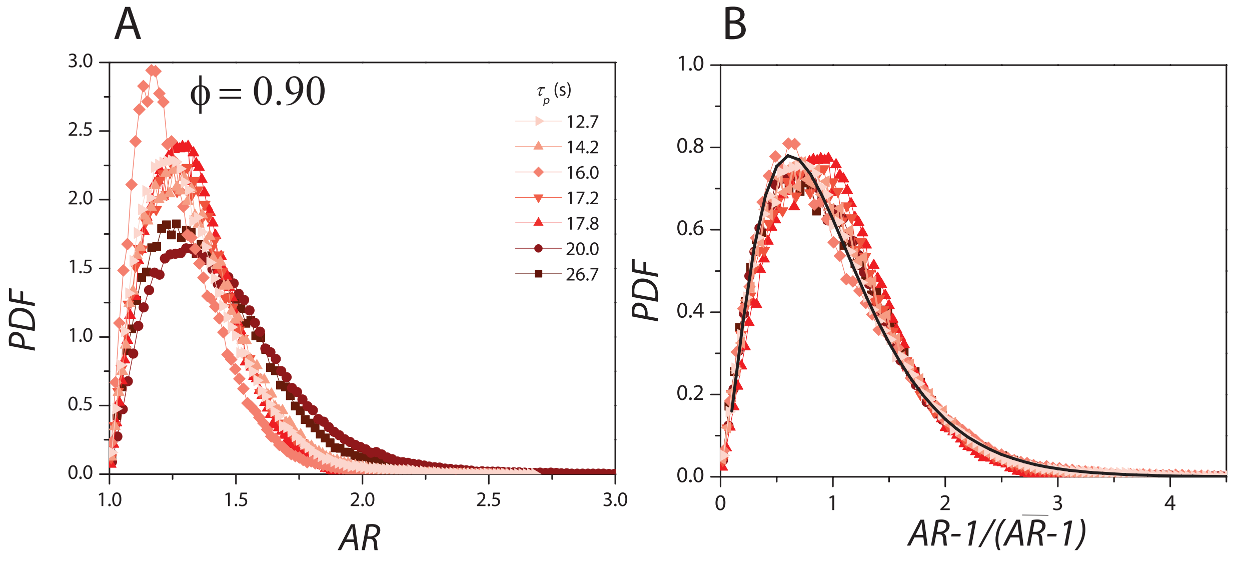

Supplementary Fig. 10: **(A)** Probability density functions (PDFs) of AR for different τ_p values for $\phi = 0.90$. **(B)** When scaled by $(AR-1)/(\overline{AR}-1)$, the PDF at different τ_p values collapse. The black line represents a $k-$Gamma distribution ($k = 2.6$).

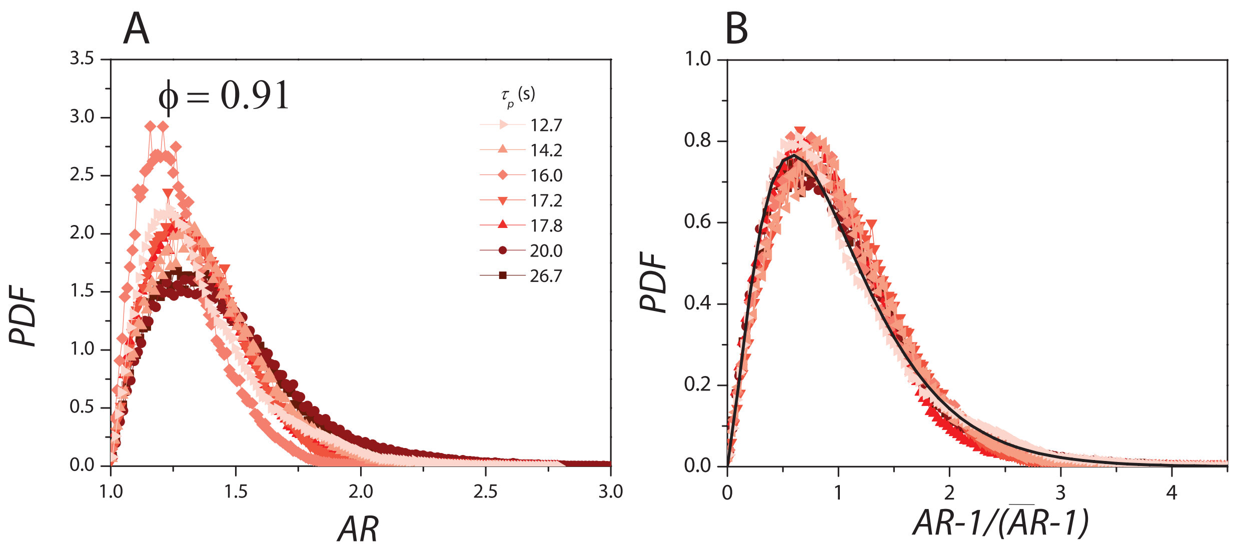

Supplementary Fig. 11: **(A)** Probability density functions (PDFs) of AR for different τ_p values for $\phi = 0.91$. **(B)** When scaled by $(AR - 1)/(\overline{AR} - 1)$, the PDF at different τ_p values collapse. The black line represents a $k-$Gamma distribution ($k = 2.4$).

D. Boundary effects

At high densities, we took specific measures to eliminate any potential effects of confinement-induced shape changes in our analysis. We excluded all granular cells in the layer making contact with the confining wall. This step was essential to ensure that the shape distribution statistics we obtained were not influenced by the confinement of cells near the boundary. To determine if the confining boundary introduces spatial gradients in cell shape changes within the imaging area, we divided the field of view into two regions: an inner radius (r_1) and an annulus (r_2-r_1) **Supplementary Fig. 12A**. These regions were chosen so that they contain approximately the same number of granular cells (38 cells) to ensure a balanced comparison. **Supplementary Fig. 12B-E** shows the probability density functions (PDFs) of the aspect ratio (AR) for packing fractions $\phi \approx 0.92$ (top panels) and $\phi \approx 0.92$ (bottom panels) and for different τ_p values. For both these values of ϕ and the intervening values, we observed a re-entrant in the dynamics (Fig. 2 b-e of the main manuscript). For the image shown in Fig. 1g of the main manuscript, the corresponding PDFs are shown in **Supplementary Fig. 12B** top panel. The statistics for calculating the PDFs are now reduced due to the fewer cells being considered in the analysis, but nonetheless, we see that the PDFs of the cells in the inner and outer rings almost overlap for all but one value of $\phi = 0.92$ and $\tau_p = 20$ s (**Supplementary Fig. 12C**, top panel). This suggests that the confining boundary does not significantly influence the dynamics.

E. Brief description of the mean-field theory of cell shape variability and the link between structure and dynamics

A recent theoretical study, Ref. [1], obtained an analytical expression for cell shape variability in confluent epithelial monolayers:

$$P(AR) = \frac{1}{\mathcal{N}} \left(AR + \frac{1}{AR} \right)^{3/2} \left(1 - \frac{1}{AR^2} \right) e^{-\alpha\left(AR + \frac{1}{AR}\right)}, \tag{5}$$

where AR gives the cellular aspect ratio, $\mathcal{N}$ is the normalization constant

$$\mathcal{N} = \Gamma(5/2)/\alpha^{5/2} - W(5/2)_1F_1(5/2, 7/2, -2\alpha), \tag{6}$$

with $W(x) = 2^x/x$, $\Gamma(x)$ is the Gamma function, and ${}_1F_1(a, b, c)$ is the Kummer's confluent Hypergeometric function. $\alpha \propto \lambda_P(1 - Kp_0)/T$ contains all the system-specific parameters with K being a constant.
Equation (5) shows that a single parameter, α, describes the cell-shape variability in diverse epithelial systems. This

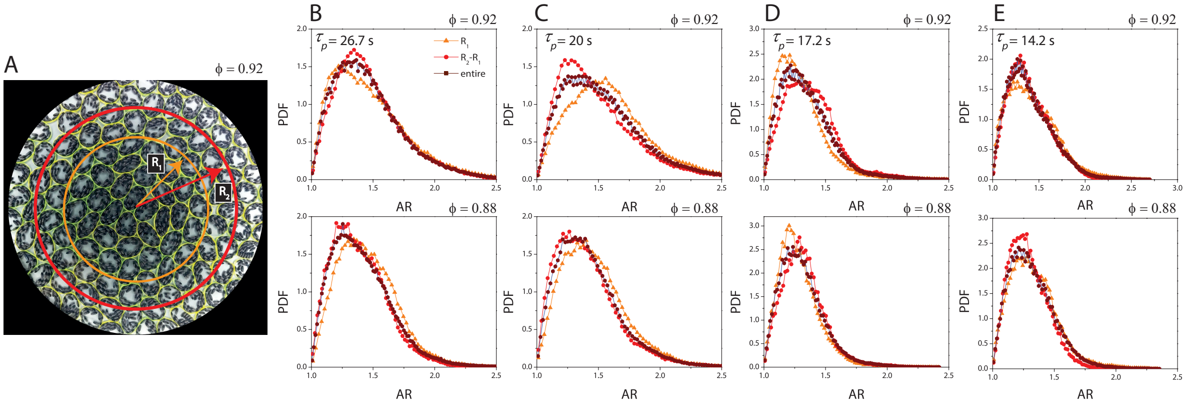

Supplementary Fig. 12: **(A)** Snapshot of a confluent assembly ($\phi \approx 0.92$). We have an equal number of cell centers within the inner circle (shown by the orange circle) and in the annular region formed by the red and orange circles. **(B-E)** Probability density functions (PDFs) of AR for cells in the inner and outer rings for different τ_p values for $\phi = 0.92$ and $\phi = 0.88$, respectively.

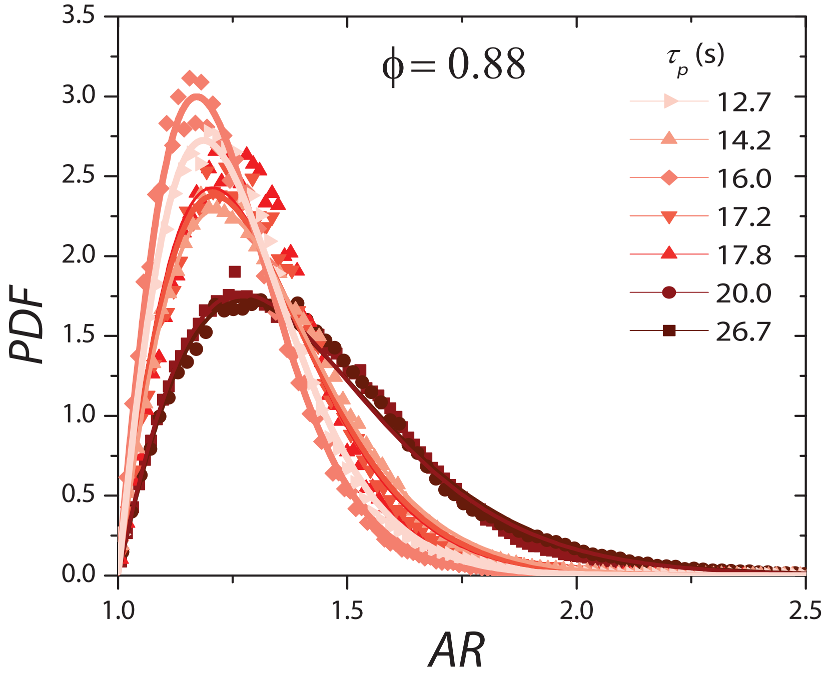

Supplementary Fig. 13: Probability density functions (PDFs) of AR for different τ_p values for $\phi = 0.88$. The solid lines are a fit to equation 5

result has two primary consequences: (1) the distribution of the rescaled variable $x = (AR - 1)/(\overline{AR} - 1)$, where $\overline{AR}$ is the average AR, becomes nearly universal, and (2) the behavior of standard deviation, SD, vs $\overline{AR}$ becomes universal.

We have tested the first result in Fig. 3b and 3c of Main Manuscript. The source of the universal relation between SD and $\overline{AR}$, the second result above, is as follows. We can use $P(AR)$ to analytically calculate both SD and $\overline{AR}$. Since $P(AR)$ depends on only one parameter α, both SD and $\overline{AR}$ must be functions of α alone. Therefore, we can use these two functions and eliminate α, writing a parametric equation for SD vs $\overline{AR}$. Since this equation does not depend on α, it must be universal, i.e., the relation cannot depend on the details of the system. We have shown the comparison of this prediction with the experimental results in Fig. 3a of Main Manuscript.

According to [1], α in Eq. 5 is a system-specific parameter predicted to correlate with dynamics. We determined

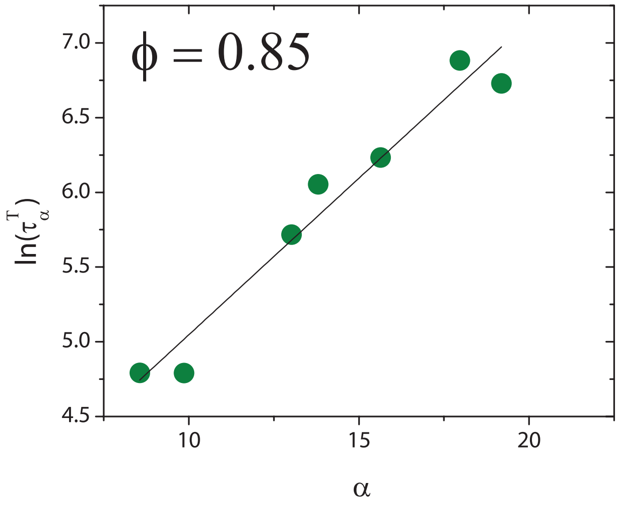

Supplementary Fig. 14: $\ln(\tau_\alpha^T)$ versus α for $\phi = 0.85$

α values by fitting the PDF of AR to Eq. 5. The solid lines in **Supplementary Fig. 13** are fits to Eq. 5.

To check whether α is correlated with the dynamics, we plotted $\ln(\tau_\alpha^T)$ versus α for a representative $\phi = 0.85$. Strikingly, as was seen in [1], $\ln(\tau_\alpha^T)$ grows linearly with α (**Supplementary Fig. 14**).

F. Quantifying dynamical heterogeneity

The translational non-Gaussian parameter, α_2^T, was used to characterize the heterogeneous dynamics. α_2^T quantifies the deviations from Gaussian dynamics and is defined as:

$$\alpha_2^T = \frac{\langle \Delta r^4(t) \rangle}{2\langle \Delta r^2(t) \rangle^2} - 1 \tag{7}$$

Here Δr is the particle displacement over time t. The time corresponding to the maximum in α_2^T is the characteristic time, namely cage breaking t^*, where the dynamics is maximally heterogeneous. We identified the top 10% translationally most-mobile particles and most-immobile particles over $\Delta t = t^*$. **Supplementary Fig. 15** shows $\alpha_2^T(t)$ for different activities for $\phi = 0.90$.

G. Numerical Methods

In the last few decades, many numerical models on confluent epithelial cell monolayer have been proposed. For example, discrete Cellular potts model (CPM) [2], continuum Vertex [3–5] and Voronoi model [6–8], Phase field models [9, 10] etc. All these models use an effective energy function given by,

$$\mathcal{H} = \sum_{i=1}^{N} \lambda_P (P_i - P_0)^2 + \lambda_A (A_i - A_0)^2 \tag{8}$$

where, N is the total number of cells, P_i and A_i are the instantaneous area and perimeter of the ith cell. The first term in Eq. (8) is a combination of the contractility of the actomyosin cortex (quadratic in P_i) and cell-cell adhesion along with cortical tension (both linear in P_i) with a strength, λ_P. The target perimeter, P_0, increases as cortical tension decreases and decreases as cell-cell adhesion increases. Tissue incompressibility in three dimensions allows us to make a $2d$ description via an area constraint i.e. second term of the energy function. Cells try to achieve a target area A_0 by adjusting their height. It is a result of the monolayer's resistance to height fluctuations or cell bulk elasticity, where λ_A is a height elasticity and A_i is the cross-sectional area (apical) of cell i and A_0 is the preferred area for the cell. For the scope of this work, we have used CPM and Vertex models described below;

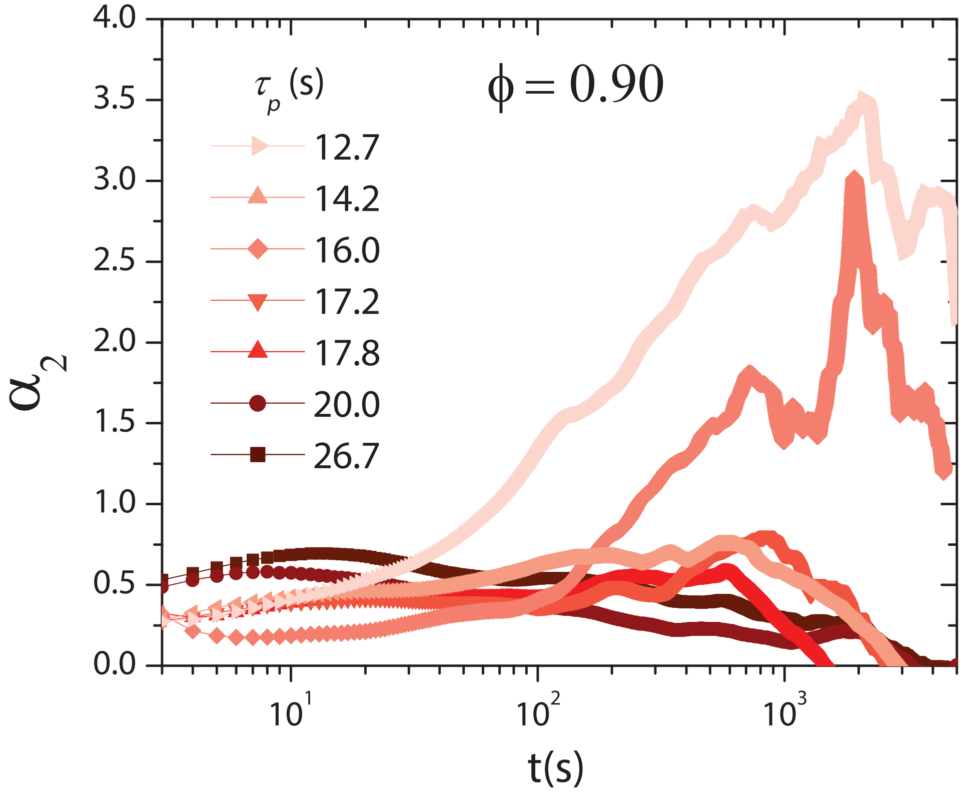

Supplementary Fig. 15: The translational non-Gaussian parameter $\alpha_2^T(t)$ for various $|\chi_{\mathrm{cell}}|$ values for $\phi = 0.92$. The time corresponding to the peak value of $\alpha_2^T(T)$ is $t^\star$.

1. *Cellular potts model*

Within the Cellular Potts model, the cell monolayer is represented as a $L \times L$ lattice. On each pixel of the lattice a Potts variable, $\sigma \in [1, N]$, is assigned, where N is the total number of cells. Unless specified, we have simulated 360 cells. A cell is represented as the collection of lattice pixels with the same Potts variable. Due to the discrete nature of the lattice structure, CPM allows for irregular cell boundaries. Unlike the Vertex model, neighbor exchange processes like topological $T1$ transitions are naturally included within the model. For the distribution of the aspect ratio of cells, simulation parameters are as follows: $\lambda_A = 1.0$, $\lambda_P = 0.25$, $A_0 = 40$, and $P_0 = 26$. Before collecting the data, we have equilibrated the system for 10^6 time steps. For the simulation of the "mimicking dynamical heterogeneities", we have used the software CompuCell3D [11]. The parameters used in CompuCell3D are : $\lambda_A = 1.0$, $\lambda_P = 0.25$, $A_0 = 100$, $T = 25$ and $P_0 = 38$ with total number of cell, $N = 100$. Before collecting the data, we have equilibrated the system for 5×10^5 time-steps.

2. *Vertex Model*

In the Vertex model (VM), cells are represented as polygons. The degrees of freedom in VM are the vertices. The vertices of the cells move stochastically by a certain distance, δr using Eq. (8) via Monte Carlo algorithm [12]. We have discretized the continuum configuration in VM into a 500×500 grid to calculate the aspect ratio of the cells accurately. Unless specified, we have simulated a binary mixture ($A_{0\alpha} = 1.2$ and $A_{0\beta} = 0.8$ such that the average area remains 1) of total $N = 400$ cells with $p_0 = 3.75$. Other parameters are as follows: $T = 0.009$, $\lambda_P = 0.02$, $\lambda_A = 0.5$.

Both the cellular Potts model (CPM) and the Vertex model (VM) use the same energy function, Eq. (8). We can always divide the energy function, $\mathcal{H}$, by a constant as it does not affect the behavior of the system. Thus, the value of λ_A only sets the energy scale. We have set $\lambda_A = 1.0$ for the CPM and $\lambda_A = 0.5$ for the VM following conventions from previously published works.

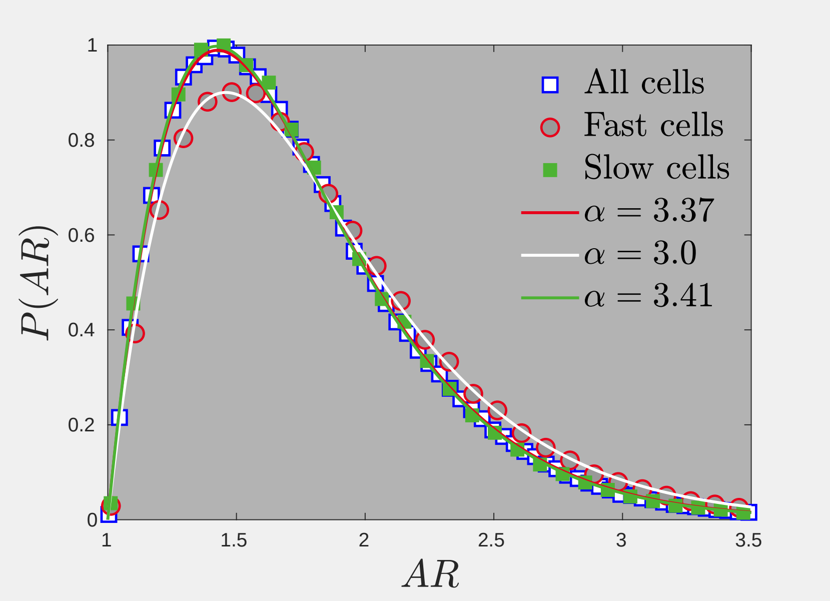

Supplementary Fig. 16: PDF of AR for all cells (hollow squares), 10 % fast (red circles) and 10 % slow cells (solid squares) from CPM simulation at $T = 25$.

The value of $\sqrt{A_0}$ sets the length scale. Existing works on the VM show a rigidity transition with P_0. The values of A_0 sets the scale for P_0. We have used P_0 such that the system is in the glassy regime as our system shows glassy dynamics. λ_P sets the scale of fluctuation. For a given value of λ_P, we can choose T for specific behavior. We showed in Ref. [13] that the qualitative behavior does not depend on the value of λ_P. Finally, we chose the values of T in the two models such that the dimensionless relaxation times in simulations are similar to that in the experiments, of the order of $500s$. However, we have also simulated the systems at other values of T and found that the qualitative results remain similar.

H. Fast cells violate mean-field predictions

In Fig. 4C of the Main Manuscript we noted that for the slow cells, the growth of $SD(AR)$ with $\overline{AR}$ follows the universal scaling; however, for the fast cells, surprisingly, it lies below the predicted line, implying that these cells show reduced shape variability.

To assess the extent to which fast and slow cells agree with the predicted scaling $SD(AR) = 0.71\overline{AR} - 0.75$, we performed a linear regression analysis. First, we fitted straight lines to $SD(AR)$ versus $\overline{AR}$ for the fast (F) and slow (S) cells from experiments and simulations separately and then calculated the sum of squared errors ($SSE_{\mathrm{F,S}}$) between the best-fit lines and the predicted scaling. Here, $SSE_{\mathrm{F,S}} = \sum_{i=1}^{N}(SD(AR)^{\mathrm{F,S}}_{\mathrm{Fit}} - SD(AR)_{\mathrm{Th}})^2$, and N is the total number of data points in each of the experimental/simulation data sets. The results of this analysis for the experiments and simulations are now summarized in **Supplementary Fig. 18**. We observed that in all sets of data, $SSE_{\mathrm{F}} > SSE_{\mathrm{S}}$, and in some instances, by almost an order of magnitude. This analysis unambiguously reveals that fast cells veer off mean-field theoretical predictions and strengthen our observations.

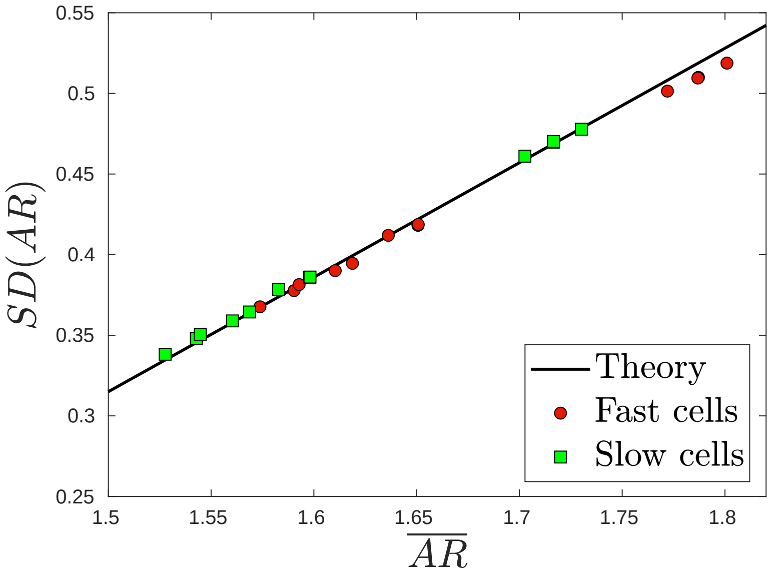

Supplementary Fig. 17: $SD(AR)$ versus $\overline{AR}$ for all cells, fast and slow cells obtained from CPM simulations.

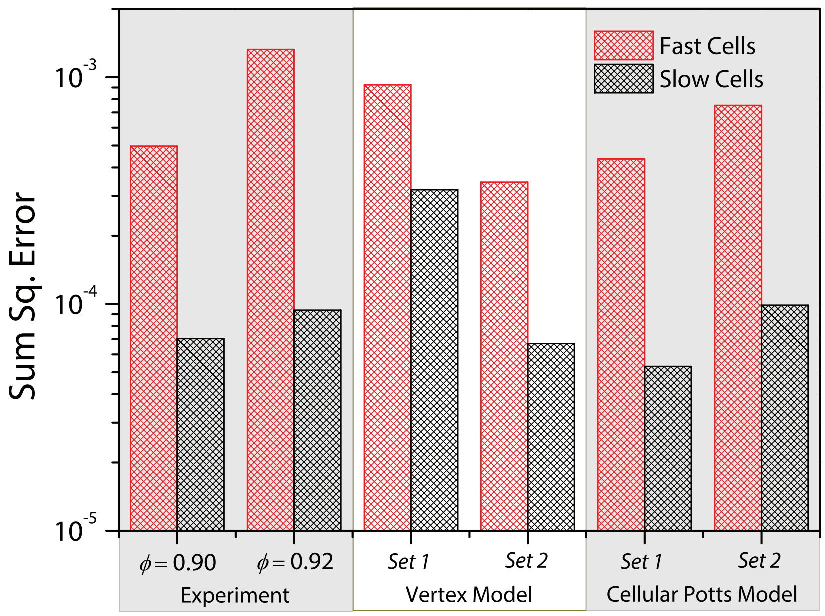

Supplementary Fig. 18: The sum of squared errors for fast and slow cells for experimental data sets at $\phi = 0.92$ and $\phi = 0.90$, as well as for the Vertex Model and the Cellular Potts Model data sets.

III. SUPPLEMENTARY REFERENCES

[1] Sadhukhan, S., & Nandi, S. (2022). On the origin of universal cell shape variability in a confluent epithelial monolayer. Elife, 11, e76406.

[2] Graner, F., & Glazier, J. A. (1992). Simulation of biological cell sorting using a two-dimensional extended Potts model. Physical review letters, 69(13), (2013).

[3] Farhadifar, R., Röper, J., Aigouy, B., Eaton, S. & Jülicher, F. The Influence of Cell Mechanics, Cell-Cell Interactions, and Proliferation on Epithelial Packing. *Current Biology*. **17**, 2095-2104 (2007)

[4] Staple, D., Farhadifar, R., Röper, J., Aigouy, B., Eaton, S. & Jülicher, F. Mechanics and remodelling of cell packings in epithelia. *The European Physical Journal E*. **33**, 117-127 (2010,10)

[5] Fletcher, A., Osterfield, M., Baker, R. & Shvartsman, S. Vertex Models of Epithelial Morphogenesis. *Biophysical Journal*. **106**, 2291-2304 (2014)

[6] Bi, D., Yang, X., Marchetti, M. & Manning, M. Motility-Driven Glass and Jamming Transitions in Biological Tissues. *Phys. Rev. X*. **6**, 021011 (2016,4)

[7] Paoluzzi, M., Angelani, L., Gosti, G., Marchetti, M., Pagonabarraga, I. & Ruocco, G. Alignment interactions drive structural transitions in biological tissues. *Phys. Rev. E*. **104**, 044606 (2021,10)

[8] Sussman, D. & Merkel, M. No unjamming transition in a Voronoi model of biological tissue. *Soft Matter*. **14**, 3397-3403 (2018)

[9] Najem, S. & Grant, M. Phase-field model for collective cell migration. *Phys. Rev. E*. **93**, 052405 (2016)

[10] Zhang, G. & Yeomans, J. Active Forces in Confluent Cell Monolayers. *Phys. Rev. Lett.*. **130**, 038202 (2023)

[11] Swat, M., Thomas, G., Belmonte, J., Shirinifard, A., Hmeljak, D. & Glazier, J. Multi-Scale Modeling of Tissues Using CompuCell3D. *Computational Methods In Cell Biology, Methods in Cell Biology*. **110** pp. 325-366 (2012). PMID:22482955 PMCID: PMC3612985 DOI: doi.org/10.1016/b978-0-12-388403-9.00013-8

[12] Wolff, H.B., Davidson, L.A. & Merks, R.M.H. Adapting a Plant Tissue Model to Animal Development: Introducing Cell Sliding into VirtualLeaf. *Bull Math Biol*. **81**, 3322–3341 (2019)

[13] Sadhukhan, S., & Nandi, S. (2022). Theory and simulation for equilibrium glassy dynamics in cellular potts model of confluent biological tissue. Phys. Rev. E, 103:062403.
