# Supplementary material for: A shape-driven reentrant jamming transition in confluent monolayers of synthetic cell-mimics
Source: Nat Commun. 2024 Jul 5;15:5645. doi: 10.1038/s41467-024-49044-z (PMC11226658; doi:10.1038/s41467-024-49044-z)
Supplement: Supplementary file 3 — Description of Additional Supplementary Files [file 41467_2024_49044_MOESM3_ESM.docx]

Description of Additional Supplementary Files

**File Name: Supplementary Movie 1**

Vertically driven achiral polar active ellipsoids confined within paper rings.

The movie shows vertically driven achiral polar active ellipsoids enclosed within a paper ring. The interplay of membrane curvature and particle orientation resulted in their accumulation at diametrically opposite ends, with their polarity pointing outwards. This accumulation not only reduced cell movement but also resulted in non-uniform stiffness. The movie plays 3X faster than in real-time.

**File Name: Supplementary Movie 2**

Deformable granular cells with tunable activity

The movie shows membranes enclosing *N*=20 chiral active ellipsoids but with different magnitudes of chirality, *χ*_Cell_, within their interior. Changing *χ*_Cell_ helps tune cell activity.

The movie plays 20x faster than in real-time.

**File Name: Supplementary Movie 3**

Unidirectional collective edge flow

The movie shows that the total system chirality, *χ*_Sys_, has a qualitative effect on the dynamics. We observed an emergent edge current in a nearly confluent assembly (*φ*=0.94) of all clockwise spinning cells, i.e., *χ*_Sys_ =1. This edge current is fully suppressed when *χ*_Sys_ =0; see Movie 4.

The movie plays 400x faster than in real-time.

**File Name: Supplementary Movie 4**

Mimicking a confluent cell monolayer

To mimic a confluent cell monolayer with no edge current, we set *χ*_Sys_ = 0 by having an equal number of clockwise (green) and counterclockwise (yellow) spinning cells on the plate. The movie demonstrates that the edge flows are absent in these assemblies for *φ*=0.92

The movie plays 400x faster than in real-time.

**File Name: Supplementary Movie 5**

Clustering of granular cell assemblies on increasing activity

Movies of the granular cell assemblies for *φ*=0.44 for three representative activities. Note a weak tendency of the cells to form clusters with increasing *τ_p_*

The movie plays 25x faster than in real-time.

**File Name: Supplementary Movie 6**

Dynamics of membranes at confluence.

This movie shows the dynamics of three representative *τ_p_*  values at confluence *φ*=0.92. Notably, increasing *τ_p_* resulted in a re-entrant behavior: structural relaxation was fastest at an intermediate value of *τ_p_*.

The movie plays 400x faster than in real-time.

**File Name: Supplementary Movie 7**

Particle displacement maps and dynamical heterogeneities.

Movie showing displacement maps over *t** for four representative *τ_p_*  values at *φ*=0.92. The color bar shows mobility, with red representing high mobility and blue representing low mobility. The arrows represent the displacement direction of individual cells. The red circles represent the top 10% translationally most mobile particles over a time interval of *t*.*

The movie plays 100x faster than in real-time.
